# CircN4bp1 Facilitates Sepsis-Induced Acute Respiratory Distress Syndrome through Mediating Macrophage Polarization via the miR-138-5p/EZH2 Axis

**DOI:** 10.1155/2021/7858746

**Published:** 2021-12-30

**Authors:** Dongyang Zhao, Chunxue Wang, Xiandong Liu, Na Liu, Shougang Zhuang, Qianqian Zhang, Xiaowei Bao, Shumin Xu, Xiaohui Zhou, Qinshu Meng, Shao Li, Lunxian Tang

**Affiliations:** ^1^Department of Internal Emergency Medicine and Critical Care, Shanghai East Hospital, Tongji University School of Medicine, Shanghai 200120, China; ^2^Department of Nephrology, Shanghai East Hospital, Tongji University School of Medicine, Shanghai, China; ^3^Department of Medicine, Rhode Island Hospital and Alpert Medical School, Brown University, Providence, RI, USA; ^4^Research Center for Translational Medicine, Shanghai East Hospital, Tongji University, Shanghai 200120, China; ^5^Department of VIP Clinic, Shanghai East Hospital, Tongji University School of Medicine, Shanghai, China

## Abstract

We recently reported the differential circRNA expression patterns of the pulmonary macrophages in sepsis-induced acute respiratory distress syndrome (ARDS) mice model by microarray analysis. However, their function and hidden molecular mechanism in regulation of macrophage activation and inflammation remain poorly understood. In this study, we found that circN4bp1was overexpressed in PBMC and monocytes, and its expression levels were correlated with a poor prognosis in sepsis induced ARDS patients induced by sepsis. Knockdown of circN4bp1 inhibited the lung injury and improved the long-time survival through blunting the M1 macrophage activation in cecal ligation and puncture- (CLP-) induced ARDS mice. Moreover, bioinformatics analysis predicated a circN4bp1/miR-138-5p ceRNA network, which was confirmed by luciferase reporter assay and RNA binding protein immunoprecipitation (RIP). CircN4bp1 affected macrophage differentiation by binding to miR-138-5p, thus regulating the expression of EZH2 in vivo and ex vivo. Lastly, the m6A level of circN4bp1was found to be elevated in ARDS mice; inhibition of m6A methyltransferase METTL3 blocked this response in vitro. Therefore, circN4bp1 can function as a miR-138-5p sponge for the modulation of macrophage polarization through regulation the expression of EZH2 and may serve as a potential target and/or prognostic marker for ARDS patients following sepsis.

## 1. Introduction

Sepsis is defined as progressive, inflammatory responses to overwhelming infections, and multiorgan dysfunction is the major cause of death. Acute respiratory distress syndrome (ARDS) remains the most common severe sepsis complications with no recommended standard treatments thus far [1, 2]. Recently, increasing evidence has shown that macrophages are key players in the pathogenesis of ARDS induced by sepsis [3, 4], which can switch from an initial proinflammatory M1 phenotype during the phase of onset of lung injury to an anti-inflammatory M2 phenotype with initiation of lung repair stage [4]. We and other investigators have recently provided direct evidence documenting the ability of in vivo and ex vivo polarized M2 phenotypes to protect against the lung injury, inflammation, and subsequent fibrosis of ARDS ^[5.6]^. However, the underlying mechanisms regulating the gene expression in the response of macrophages to the inflammatory microenvironment are still unclear.

Circular RNAs (circRNAs) are a class of newly identified noncoding RNAs and are produced from precursor mRNA back-splicing by covalently closed, single-stranded RNA circles at junction site of 3′,5′-phosphodiester bonds. Several studies have highlighted the regulatory mechanisms by which circRNAs participate in gene regulation, functioning as miRNA “sponge” to sequester and competitively suppress miRNA activity [5], regulating transcription of genes and translating protein genes [5]. Their interactions with disease-associated miRNAs indicate that circRNAs are crucial in a variety of diseases and might serve as novel diagnostic biomarkers and therapeutic targets [6, 7]. However, the role of circRNAs in the gene expression occurring during macrophage polarization is still less clear. Only recently, a report documented the general changes of circRNAs induced by the activation of primary bone marrow-derived macrophages (BMDMs) under two distinct polarizing conditions (M1 and M2 macrophages) in vitro [8]. Furthermore, transfection with the circHECTD1 lentivirus attenuated the phenotypic transformation of the macrophages treated with SiO2 [9], while circPPM1F modulates M1 macrophage activation in type 1 diabetes mellitus [10]. We have recently reported the global changes in the circRNA expression patterns and circRNA-miRNA-mRNA networks of the pulmonary macrophage activation in a typical cecal ligation and puncture- (CLP-) induced ARDS mice model by microarray analysis, suggesting that circRNAs might make a vital contribution to the macrophage differentiation and the development of ARDS [11]. However, the detailed role and molecular mechanism of circRNAs in macrophage polarization and sepsis-induced ARDS remain to be documented. Furthermore, accumulating evidence indicates that N6-methyladenosine (m6A) is the most universal epigenetic modification on mRNAs and noncoding RNAs (ncRNAs) in eukaryotes and is essential for multiple RNA processing events in physiological and pathological processes [12]. CircRNAs can be m6A-modified in way that is distinct from those of mRNAs [12]. To date, the contributions of m6A modification for circRNAs in ARDS have not been elucidated.

Based on previous data from our laboratory [11], circN4bp1 (chr8: 86865721-86866760) was of particular interest because one of its most likely targeted gene, EZH2 (a histone methyltransferase), had been previously documented by our team to be involved in sepsis-induced inflammation and lung injury through modulating macrophage M1 polarization [13]. Hence, in the present study, we explored the function and underlying mechanisms of circN4bp1 in sepsis-induced ARDS in vivo and in vitro.

## 2. Materials and Methods

### 2.1. Patients, Controls, and Animals

Between January 2020 and February 2021, a total of 40 eligible patients, with an intensive care unit (ICU) length of stay ≥ 48 h, were classified as sepsis according to the Surviving Sepsis Campaign definitions [14] and then were followed up for development of acute respiratory distress syndrome (ARDS) based on the Berlin definition [15] from the emergency and/or general intensive care unit (ICU) of East Hospital, Tongji University (Shanghai, China). For comparison, peripheral blood was also taken from 40 age- and gender-adjusted healthy volunteers who came to East hospital for routine physical examination. The characteristics of the participants are shown in supplementary, Table S1. The following information was collected and recorded: demographic characteristics (age and sex), Sepsis etiology, admission source, respiratory index (tidal volume, PaO2/FiO2 ratio at baseline, PEEP, oxygenation index), incidence of shock, and modified Sequential Organ Failure Assessment (mSOFA) score. All the patients were followed by 28 days and divided into survival and nonsurvival groups according to whether they were alive or not. This study was approved by the Research Ethics Board of East Hospital, Tongji University (Shanghai, China). All recruited patients or their authorized family members were given a written consent document. We isolated peripheral blood mononuclear cells (PBMC) from patients and controls according to the protocol as previously reported [13]. PBMCs and serum samples are also stored at −20°C for subsequent use. In addition, CD14+, CD3+, and CD19+ cells were sorted from PBMCs with a magnetic cell sorting system (Miltenyi Biotec, Germany). Sorted cells were subjected to RNA extraction and qRT-PCR.

Male C57BL/6 mice (Shanghai Super-B&K Laboratory Animal Corp. Ltd., Shanghai, PR China) aged 6–8 weeks (16–18 g) were housed in a pathogen-free facility at Tongji University to construct sepsis-induced ARDS mice model by Cecal ligation and puncture (CLP) surgery as previously reported [13]. All animal experiments were performed according to the guidelines for the Care and Use of Laboratory Animals (Ministry of Health, China, 1998). Experiments were conducted under protocols approved by the Animal Use Committee of East Hospital/Tongji University. The mice were randomly divided into four experimental groups: sham, sham+circN4bp1-KD, CLP+ vector, and CLP+ circN4bp1-KD group. In the treatment study, mice are randomly separated into the si-circRNA-treated Raw264.7 macrophage group and sh-NC-treated macrophage group (*N* = 12). Mice were intravenously injected with plasmid transfected macrophages through the tail vein 24 hours before CLP. Mortality was recorded for up to 10 days postprocedure. For the nonsurvival experiment, mice were sacrificed 24 h after CLP. Blood samples were collected by cardiac puncture, and lung tissues were harvested for histological analysis, which were fixed in 10% buffered formalin as previously reported [13]. Analysis of BALF was conducted and wet/dry weight ratio was calculated as we previously described [13]. Macrophages were isolated from BALF and lung tissue homogenates according to our previously reported methods [13, 16]. All samples and isolated cells are stored at −20°C for determinations.

### 2.2. RNA Extraction and Quantitative Real-Time PCR (qRT-PCR)

The nuclear and cytoplasmic fractions or total RNA were extracted using TRIzol (Invitrogen, USA) followed by reverse transcription of mRNAs and circRNAs using PrimeScript II 1st Strand cDNA Synthesis Kit (Takara, Japan) according to the standard manufacturer's instructions. A qRT-PCR assay was performed to measure mRNAs and circRNA expression with SYBR® Premix Ex Taq™ II (Takara, Japan) using the Roche 480 Real Time PCR System. A GAPDH endogenous control was used for normalization. Relative quantification (2^−*ΔΔ*CT^) was used for result analysis. All the primers used were listed in supplementary, Table S2.

### 2.3. Cell Culture and Transfection

MH-S cells, the SV40 transformed mouse alveolar macrophage cell line (CRI-2019, ATCC, Baltimore, Md, USA), and Raw264.7 cells were cultured in RPMI-1640 medium (Gibco, CA, USA) containing 10% fetal bovine serum (Gibco, Australia Origin) and 1% penicillin/streptomycin in an atmosphere of 5% CO_2_ and 95% air at 37°C. According to the previous report [16], MH-S and Raw264.7 cells were polarized as M1 or M2 macrophages with the indicated stimulants: 50 ng/ml LPS (L3024, Sigma, Mo) 24 h for M1 polarization and 10 ng/ml IL-4 (404-ML, R&D Systems) 24 h for M2 polarization. CircN4bp1 overexpressed vector (pc-circN4bp1, 2 *μ*g), circN4bp1 silence vectors (sh-circN4bp1, 2 *μ*g), miR-138-5p mimics (50 pmol), miR-138-5p inhibitor (50 pmol), and the corresponding control vectors were constructed by GenePharma (Shanghai, China). MH-S cells and Raw264.7 cells were transfected with the above vectors by Lipofectamine 2000 (Invitrogen) according to the manufacturer's instructions 24 h before LPS or IL-4 stimulation. Stably transfected cells are finally verified by quantitative real-time PCR. After different stimulations, cells and supernatants were harvested for analyses. All the in vitro experiments were repeated at least three times.

### 2.4. Fluorescence In Situ Hybridization (FISH) Assay

The location of circN4bp1 in macrophages is determined by FISH. Macrophages are fixed with 4% paraformaldehyde and gradient dehydrated with ethanol. Fluorescent-labeled probe for circN4bp1 is applied during hybridization. We use DAPI (Beyotime, Shanghai, China) to stain the nucleus of macrophages.

### 2.5. Luciferase Reporter Assay

Raw264.7 cells are used to estimate the targets of circN4bp1 and miR-138-5p. Cells were transfected with miRNA mimics, inhibitors, circRNA-WT, circRNA-MUT, or the corresponding plasmids with lipofectamine 2000. Cells were lysed, and the luciferase activity was determined by Picagene Dual SeaPansy luminescence kit (Toyo Inc., Japan) based on the manufacturer's instructions as reported [17].

### 2.6. RNase *R* Digestion RNA Stability

Four micrograms total RNA from RAW264.7 cells was either untreated (control) or treated with 20 units of RNase *R* (Epicenter; USA, RNR07250) in the presence of 1× reaction buffer and incubated for 30 min at 37°C. The digested RNA was isolated using acid phenol-chloroform (5 : 1). Then, reverse transcription and qRT-PCR were performed, as described in the RNA extraction and qRT-PCR section. RAW264.7 cells (1 × 10^5^) were placed in 24-well plates and treated with 250 ng/ml actinomycin D (Act D, Sigma) added to the cell culture medium. The levels of circN4bp1 and N4bp1 were detected at 0, 8, 12, and 24 h.

### 2.7. CircRNA Immunoprecipitation (circRIP) Assay

Biotin-labeled circN4bp1 probe was synthesized by GenePharma (Shanghai, China), and a circRIP assay was performed as previously described [17]. Briefly, Raw264.7 cells were washed with ice cold PBS, fixed using formaldehyde, lysed in the co-IP buffer, and sonicated. After centrifugation, the supernatant was combined with streptavidin Dynabeads M-280 (Invitrogen, Waltham, MA, USA) and incubated at 30°C for 12 h. Next, the probe dyna bead-circRNA mixture was washed and incubated with lysis buffer and proteinase *K*. Finally, the mixture was combined with TRIzol Reagent (Invitrogen, Carlsbad, CA, USA) for RNA extraction and detection.

### 2.8. ELISA Analyses

ELISAs were performed to measure concentrations of TNF-*α*, IL-6, and IL-10 protein on supernatants of lung homogenates, BALF, and cell culture supernatants, which were performed in accordance with the manufacturer's instructions (R&D Systems).

### 2.9. Immunoblotting Analysis

Immunoblotting analysis was conducted as described previously [13, 16]. Densitometry analysis of immunoblot results was conducted by using ImageJ software. Data are expressed as mean ± standard error of mean (SEM) of three replicated experiments (primary antibodies are listed in supplementary material, Table S3).

### 2.10. MeRIP-qRT-PCR

Total RNA was isolated from cultured macrophages by TRIzol. Poly (A) + RNA was purified by the GenElute mRNA Miniprep Kit. Poly (A) + RNA was fragmented using RNA fragmentation kit (Ambion, CA, USA) referring to manufacturer's instructions. Subsequently, the fragmented RNA was incubated with m6A antibody for immunoprecipitation according to the manufacturer's procedure of Magna MeRIP™ m6A Kit (Merck Millipore, USA). Enrichment of m6A containing RNA was examined by qRT-PCR.

### 2.11. Statistical Analysis

All experimental data are presented as the means ± SEM. The two-tailed Student *t*-tests were used for comparisons between two groups, and one-way analysis of variance (ANOVA) was used for multifactorial comparisons. The relationship between circN4bp1 and mSOFA was tested using Pearson's correlation and linear regression. Statistical analyses were performed with SPSS 20.0 software (SPSS Inc., Chicago, IL, USA) or GraphPad Prism 8.0 (GraphPad Software, La Jolla, CA, United States). A value of *P* < 0.05 was considered to indicate a statistically significant difference.

## 3. Results

### 3.1. Expression and Characterization of circN4bp1 in Macrophages

**Figure 1:**
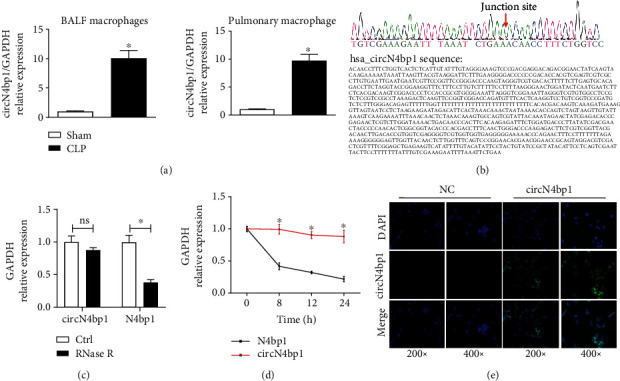
Expression and characterization of circN4bp1 in macrophages. (a) qRT-PCR of quantification of the circN4bp1 expression in the BALF and pulmonary macrophages isolated from lung tissue homogenates of sham and CLP mice. (b) Sanger sequencing showing the “head-to-tail” splicing of circN4bp1 in macrophages. (c) qRT-PCR of quantification of the circN4bp1 and N4bp1 mRNA expression in RAW264.7 macrophages after treatment with RNase *R*. (d) qRT-PCR quantification of the circN4bp1 and N4bp1 mRNA expression in RAW264.7 macrophages after treatment with actinomycin D. (e) RNA FISH for circN4bp1. Nuclei were stained with DAPI. Data are presented as means ± SD; significant difference was identified with Student's *t*-test. ^∗^*P* < 0.05; ns: no significant.

We first verified the upregulation of circN4bp1 in macrophages of the BALF and lung tissue by qRT-PCR (Figure 1(a)). Then, the sequence of circN4bp1 was confirmed with Sanger sequencing assays (Figure 1(b)). Next, we tested the property of circN4bp1 by RNase *R* treatment, as shown in Figure 1(c), and linear N4bp1 was mostly digested, while circN4bp1 remained almost unchanged, thus suggesting a circular configuration. Additionally, the half-life of circN4bp1, its expression level, together with the N4bp1 level was examined following actinomycin D transcriptional inhibition. We found that circN4bp1was more stable than N4bp1 (Figure 1(d)). Lastly, we determined the subcellular localization of circN4bp1 by conducting FISH assay and found that circN4bp1 was mainly localized in the cytoplasm of macrophages (Figure 1(e)).

### 3.2. Upregulation of CircN4bp1 Correlates with Poor Prognosis of Sepsis-Induced ARDS Patients

**Figure 2:**
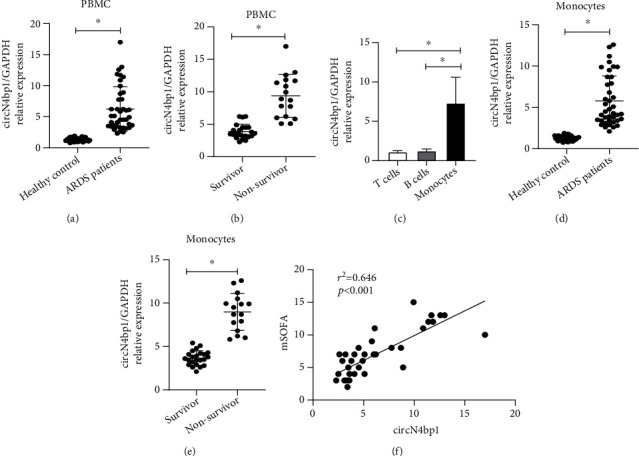
Expression of circN4bp1 in peripheral blood mononuclear cells (PBMCs). (a) qRT-PCR quantification of circN4bp1 in the PBMC of 40 ARDS patients postsepsis and 40 healthy controls. (b) qRT-PCR quantification of circN4bp1 in the PBMC of survivor and nonsurvivor group of ARDS patients postsepsis. (c) qRT-PCR assay was used to measure the circN4bp1 expression in monocytes, T cells, and B cells in PBMCs. (d) qRT-PCR quantification of circN4bp1 in the monocytes of 40 ARDS patients postsepsis and 40 healthy controls. (e) qRT-PCR quantification of circN4bp1 in the monocytes of survivor and nonsurvivor group of ARDS patients with sepsis. (f) Correlation analysis of mSOFA and the expression of circN4bp1 in monocytes from ARDS patients with sepsis (*N* = 40, Pearson's correlation). Data are presented as means ± SD; significant difference was identified with Student's *t*-test. ^∗^*P* < 0.05.

The expression levels of circN4bp1 of 40 sepsis-induced ARDS patients and 40 healthy controls were quantified using qRT-PCR. We found that circN4bp1expression levels were significantly upregulated in the PBMCs of ARDS patients relative to the controls (Figure 2(a)). Furthermore, circN4bp1was found to be significantly higher in nonsurvivors than survivors (Figure 2(b)). To assess the role of circN4bp1 in the regulation of subtypes of PBMCs, we isolated PBMCs into T cells, B cells, and monocytes to detect the expression levels of circN4bp1. We observed that circN4bp1was mainly expressed in monocytes rather than T and B cells (Figure 2(c)). Furthermore, the higher expression of circN4bp1 in monocytes was found in ARDS patients than healthy controls, with much higher levels in nonsurvivors than those survived (Figures 2(d) and 2(e)). The Spearman correlation analysis was conducted to further evaluate the prognostic value of circN4bp1in monocytes. Interestingly, the circN4bp1 expression levels in monocytes of the ARDS group were positively correlated with mSOFA score (*r* = 0.646, *P* < 0.001) (Figure 2(f)). Therefore, these findings suggested that circN4bp1 upregulation in the PBMCs and monocytes was correlated with a poor prognosis in ARDS patients, and that monitoring overexpression of circN4bp1 might predict poor outcomes of these patients.

### 3.3. CircN4bp1 Is Involved in Promoting M1 Macrophage Polarization While Inhibiting M2 Activation in Two Ex Vivo Macrophage Cell Lines

**Figure 3:**
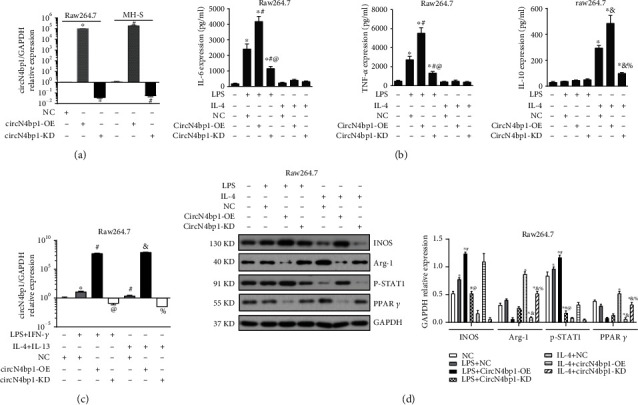
CircN4bp1 is involved in promoting M1 macrophage polarization while inhibiting M2 activation in ex vivo raw264.7 and MH-S macrophage cell lines. (a) RAW264.7 and MH-S cells were transfected with small interfering RNAs (siRNAs) targeting the circN4bp1 junction site or a circN4bp1 lentivirus plasmids. The relative expression of circN4bp1 was quantified by qRT-PCR. ^∗^*P* < 0.05 vs. control of raw264.7 cells; #*P* < 0.05 vs. control of MH-S cells. RAW264.7 was transfected with Si-circN4bp1 (circN4bp1-KD), circN4bp1 lentivirus plasmids (circN4bp1-OE), or scrambled control (NC) and then exposed to either LPS (50 ng/ml) or IL-4 (10 ng/ml) for an additional 24 h. (b) The levels of IL-6, TNF-*α*, and IL-10 were measured by ELISA in the supernatants of LPS and IL-4 stimulated raw264.7 cells. (c) The relative expression of circN4bp1 was quantified by qRT-PCR. (d) Representative western blot depicting raw264.7 cell lysates probed for iNOS, Arg-1, p-STAT1, PPAR-*γ*, and GAPDH. Expression levels of iNOS, Arg-1, p-STAT1, and PPAR-*γ* were quantified by densitometry and normalized using GAPDH. (^∗^*P* < 0.05 vs. NC group, #*P* < 0.05 vs. LPS stimulated group, ^@^*P* < 0.05 vs. circN4bp1-OE + LPS group, &*P* < 0.05 vs. IL-4 stimulated group, and %*P* < 0.05 vs. circN4bp1-OE + IL-4 group determined by one-way ANOVA for multiple group comparisons).

To determine the functional influence ofcircN4bp1on macrophage differentiation, we first specifically knocked down the expression of circN4bp1(circN4bp1-KD) in RAW264.7 and MH-S cells with small interfering RNAs (siRNAs) targeting the circN4bp1 junction site and overexpressed of circN4bp1with a circN4bp1 lentivirus plasmids (circN4bp1-OE) transfected into the two macrophage cell lines in contrast to the NC (scrambled control) group (Figure 3(a)). Next, we cultured RAW264.7and MH-S cells in the presence or absence of LPS (M1 polarization) or IL-4 (M2 phenotype) as previously reported [16] and treated them with Si-circN4bp1 or circN4bp1 lentivirus plasmids for24 h. We found that genetic knockdown of circN4bp1 significantly inhibited M1 polarization as evidenced by a downregulation of M1 marker INOS (Figures 3(c) and 3(d), supplementary Figure S1A,1B) and related cytokines as IL-6 and TNF-*α* (Figure 3(b), supplementary Figure S1C), while augmented the M2 polarization, as indicated by upregulation of M2 markerArg-1(Figures 3(c) and 3(d), supplementary Figure S1A,1B) and associated cytokine IL-10 (Figure 3(b), supplementary Figure S1C). To the contrary, the overexpression of circN4bp1 was found to promote the M1 polarization while inhibits the M2 differentiation exhibited by the increase of INOS, IL-6, TNF-*α*, and a reduction of IL-10 and Arg-1 (Figures 3(b)–3(d), supplementary Figure S1).

As we had previously documented the involvement of STAT1 pathway in M1 polarization and PPAR-*γ* in M2 differentiation [16], we further investigated the effects of circN4bp1on these pathways. Intriguingly, western blotting showed that the overexpression of circN4bp1 did indeed significantly upregulate the levels of p-STAT1 in M1 polarized macrophages while downregulates the protein levels of PPAR-*γ*. We further noticed that circN4bp1 knockdown resulted in markedly decreased levels of p-STAT1 in M1 polarized macrophages (Figures 3(c) and 3(d), supplementary Figure S1). Overall, these findings implied that circN4bp1can promote M1macrophage activation by enhancing STAT1 signaling, while inhibits the M2 macrophage polarization by suppressing PPAR-*γ* signaling pathways.

### 3.4. CircN4bp1 Functions as a Molecular Sponge for miR-138-5p

**Figure 4:**
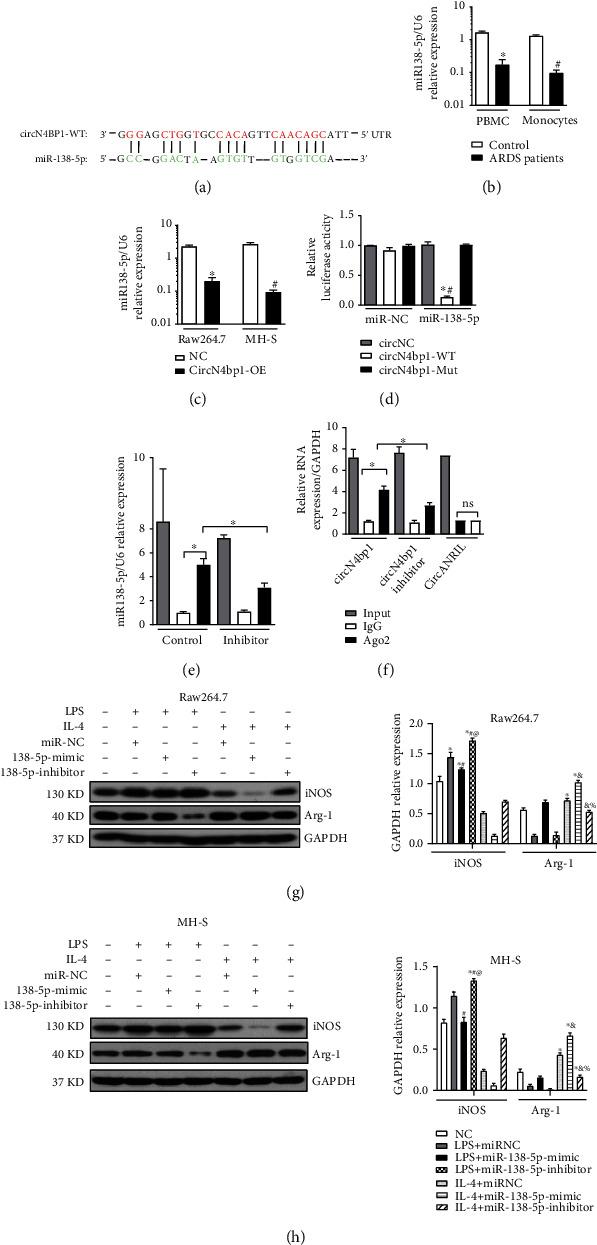
CircN4bp1 acts as a miRNA sponge for miR-138-5p. (a) Schematic showing the predicted miR-138-5p sites in circN4bp1. (b) qRT-PCR quantification of miR-138-5pin the serum and monocytes of 40 ARDS patients postsepsis and 40 healthy controls, and data are expressed as mean ± SEM (^∗^*P* < 0.05 vs. serum of healthy controls; #*P* < 0.05 vs. monocytes of healthy controls). (c) qRT-PCR quantification of miR-138-5p in the two ex vivo macrophage cell lines transfected withcircN4bp1 lentivirus plasmids (circN4bp1-OE) or scrambled control (NC), and data are expressed as mean ± SEM (^∗^*P* < 0.05 vs. scrambled control of raw264.7 cells; #*P* < 0.05 vs. scrambled control of MH-S cells). (d) Luciferase assays in raw264.7 cells cotransfected with a scrambled control, miR-138-5p mimic, and a luciferase reporter plasmid containing wild-type circN4bp1 (circN4bp1-WT) or muted-type circN4bp1 (circN4bp1-Mut). (^∗^*P* < 0.05 vs. circNC group; #*P* < 0.05 vs. circN4bp1-Mut group). (e) RIP assay was performed using input, IgG, or anti-Ago2 antibodies. Relative expression of miR-138-5p assayed by qRT-PCR. (f) RIP assay and relative expression of circN4bp1 determined by qRT-PCR. CircANRIL (a circular RNA reported not to bind to AGO2) was used as a negative control. Data of three independent assays. ^∗^*P* < 0.05. RAW264.7 and MH-S were transfected with miR-138-5p mimic or inhibitor and then exposed to either LPS (50 ng/ml) or IL-4 (10 ng/ml) for an additional 24 h. (g) Representative Western blot depicting raw264.7 cell lysates probed for iNOS, Arg-1, and GAPDH. Expression levels of iNOS and Arg-1 were quantified by densitometry and normalized using GAPDH. (h) Representative western blot depicting MH-S cell lysates probed for iNOS, Arg-1, and GAPDH. Expression levels of iNOS and Arg-1 were quantified by densitometry and normalized using GAPDH. (^∗^*P* < 0.05 vs. NC group, #*P* < 0.05 vs. LPS stimulated group, ^@^*P* < 0.05 vs. miR-138-5p mimic +LPS group, &*P* < 0.05 vs. IL-4 stimulated group, and %*P* < 0.05 vs. miR-138-5p mimic +IL-4 group determined by one-way ANOVA for multiple group comparisons).

Based on bioinformatic prediction by the miRanda and TargetScan database, miR-138-5p was considered to potentially bind with circN4bp1, and circN4bp1–miR-138-5p–mRNA network was shown in Figure 4(a). In addition, the miR-138-5p level was significantly inhibited in PBMCs and monocytes of patients with sepsis-induced ARDS patients in comparison with healthy subjects (Figure 4(b)). Based on these findings, we hypothesized that circN4bp1 could regulate macrophage function by sponging miR-138-5p. Firstly, we observed a negative association between circN4bp1 and miR-138-5p in circN4bp1-overexpressed two macrophage cell lines (Figure 4(c)). Then, a dual-luciferase assay was performed showing high binding affinity between circN4bp1 and miR-138-3p. Besides, miR-138-5p significantly reduced luciferase reporter activity when compared to the control (Figure 4(d)). A RIP assay showed that both circN4bp1 and miR-138-5p were elevated in the immunoprecipitates of the anti-Ago2 group (Figures 4(e) and 4(f)). Both circN4bp1 and miR-138-5p inhibitors were reduced in the miR-138-5p inhibitor-treated group compared with the IgG group (Figures 4(e) and 4(f)).

**Figure 5:**
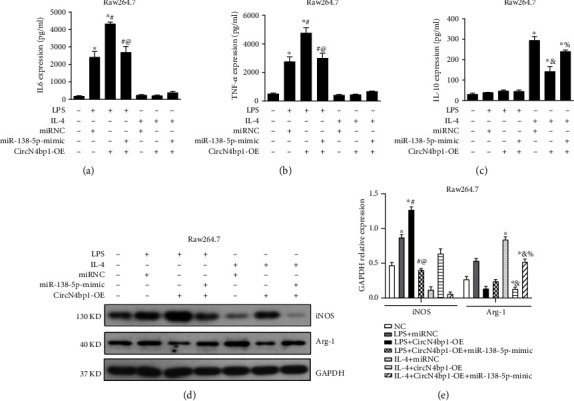
miR-138-5p mimics suppress the effect of circN4bp1 on macrophage polarization. RAW264.7 was transfected with miR-138-5p mimic with/withoutcircN4bp1 lentivirus plasmids (circN4bp1-OE) or scrambled control and then exposed to either LPS (50 ng/ml) or IL-4 (10 ng/ml) for an additional 24 h. The levels of IL-6 (a), TNF-*α* (b), and IL-10 (c) were measured by ELISA in the supernatants of LPS and IL-4 stimulated raw264.7 cells. (d) Representative western blot depicting raw264.7 cell lysates probed for iNOS, Arg-1, and GAPDH. (e) Expression levels of iNOS and Arg-1 were quantified by densitometry and normalized using GAPDH. All data are expressed as mean ± SEM. (^∗^*P* < 0.05 vs. NC group, #*P* < 0.05 vs. miRNC + LPS stimulated group, ^@^*P* < 0.05 vs. circN4bp1-OE + LPS group, &*P* < 0.05 vs. miRNC +IL-4 stimulated group, and %*P* < 0.05 vs. circN4bp1-OE + IL-4 group determined by one-way ANOVA for multiple group comparisons).

Next, we investigated the influence of miR-138-5p on macrophage differentiation in RAW264.7 and MH-S cells with miR-138-5pmimic and inhibitor. We found that miR-138-5p inhibitor could significantly promoted M1 macrophage polarization with an upregulation of INOS (Figures 4(g) and 4(h)), IL-6, and TNF-*α* (supplementary material, Figure S2A,2B,2D,2E), but a downregulation of M2 associated proteins as IL-10 (supplementary material, Figure S2C, 2F) and Arg-1 (Figures 4(g) and 4(h)) in contrast to the miR-138-5p-mimic and miR-NC group. As we had observed that circN4bp1 could promote M1 polarization while inhibit M2 activation, we further adopted the rescue tests using miRNA mimics, which showed that miR-138-5p mimics could suppress the effect of circN4bp1on macrophage polarization (Figure 5, supplementary Figure S3).

### 3.5. CircN4bp1-mir-138-5p ceRNA Modulates Macrophage Differentiation via Targeted-Regulating EZH2

**Figure 6:**
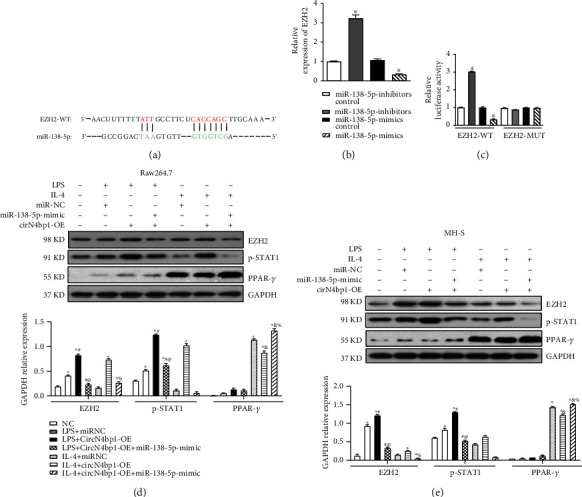
CircN4bp1-miR-138-5p ceRNA modulates macrophage differentiation via targeted-regulating EZH2. (a) Schematic showing the 3′UTR of EZH2 recognized by miR-138-5p. (b) miR-138-5p inhibited the expression of EZH2 mRNA in RAW264.7 macrophages as evidenced by qRT-PCR. (c) Luciferase reporter assay demonstrated that EZH2 was a target of miR-138-5p, but the rescue test by use of miR-138-5p inhibitors reversed its effect on EZH2. (^∗^*P* < 0.05 vs. miRNA inhibitor control group) (d) RAW264.7 cells was transfected with miR-138-5p mimic with/withoutcircN4bp1 lentivirus plasmids (circN4bp1-OE) or scrambled control and then exposed to either LPS (50 ng/ml) or IL-4 (10 ng/ml) for an additional 24 h. Representative western blot depicting raw264.7 cell lysates probed for EZH2, p-STAT1, PPAR-*γ*, and GAPDH. Expression levels of iNOS and Arg-1 were quantified by densitometry and normalized using GAPDH. (e) MH-S cells were transfected with miR-138-5p mimic with/withoutcircN4bp1 lentivirus plasmids (circN4bp1-OE) or scrambled control and then exposed to either LPS (50 ng/ml) or IL-4 (10 ng/ml) for an additional 24 h. Representative western blot depicting raw264.7 cell lysates probed for EZH2, p-STAT1, PPAR-*γ*, and GAPDH. Expression levels of iNOS and Arg-1 were quantified by densitometry and normalized using GAPDH. All data are expressed as mean ± SEM. (^∗^*P* < 0.05 vs. NC group, #*P* < 0.05 vs. miRNC + LPS stimulated group, ^@^*P* < 0.05 vs. circN4bp1-OE + LPS group, &*P* < 0.05 vs. miRNC +IL-4 stimulated group, and %*P* < 0.05 vs. circN4bp1-OE + IL-4 group determined by one-way ANOVA for multiple group comparisons).

To further investigate the downstream mRNA targets of circN4bp1-miR-138-5p ceRNA network, we performed bioinformatic analysis in the TargetScan database and found miR-138-5p could target the 3′-untranslated region (UTR) of EZH2 (Figure 6(a)). Besides, a negative association between EZH2 and miR-138-5p was found in macrophages (Figure 6(b)). The luciferase reporter assay demonstrated that EZH2 was a target of miR-138-5p, but the rescue test by use of miR-138-5p inhibitors reversed its effect on EZH2 (Figure 6(c)). Therefore, EZH2 might be the targeted gene of miR-138-5p. As we had reported that EZH2 was involved in the activation of M1 macrophage and inhibition of M2 differentiation [16], we hypothesized that circN4bp1 regulates macrophage differentiation through preventing EZH2 downregulation by miR-138-5p. To test this hypothesis, we overexpressed miR-138-5p mimics in the aforementioned two macrophage cell lines and found that the expression level of EZH2 was increased in the presence of the circN4bp1 overexpression (Figure 6(c)), whereas miR-138-5pmimics significantly inhibited the upregulation of EZH2 after the circN4bp1 overexpression (Figures 6(d) and 6(e)). CircN4bp1 could promote the expression of p-STAT1 in M1 polarization macrophages and inhibit the expression of PPAR-*γ* in M2 polarized macrophages (Figures 6(d) and 6(e)). However, miR-138-5p mimics could partially rescue this effect via cirN4bp1/miR-138-5p sponge (Figures 6(d) and 6(e)).

### 3.6. Knockdown of circN4bp1in Macrophages Alleviated Lung Injury Induced by Sepsis after CLP Surgery through Inhibition of M1 Macrophage Activation

**Figure 7:**
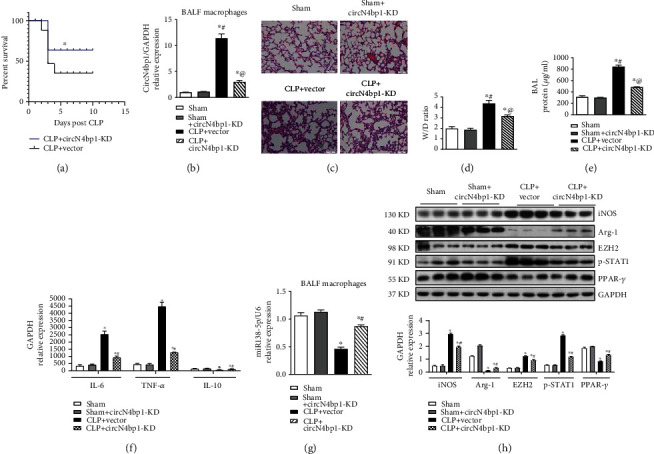
Knock down of circN4bp1 in macrophage alleviated lung injury induced by sepsis after CLP surgery through inhibition of M1 macrophage activation. (a) CLP surgery was performed on the vector (*N* = 15) and circN4bp1-KD group (*N* = 15) mice, and survival was monitored for 10 days. Mice were intravenously injected with plasmid transfected macrophages through the tail vein 24 hours before CLP. Treatment with si-circN4bp1 significantly improved long-term survival compared to the vector vehicle group. ^∗^P < 0.05, log rank test for survival study. (b) qRT-PCR of quantification of the circN4bp1expression in the macrophages isolated from BALF of the four group mice (sham, sham+circN4bp1-KD, CLP+ vector, and CLP+ circN4bp1-KD group). (c) Lungs were fixed, sectioned, and stained with H&E. Representative sections are shown for the four above mentioned groups (original magnification, ×200). (d) Evaluation of wet/dry weight ratio is compared among the four abovementioned groups. (e) Quantification of protein levels in the bronchial alveolar lavage (BAL) in the four above mentioned groups. (f) Quantification of IL-6, TNF-*α*, and IL-10 levels in the BALF macrophages in the four abovementioned groups. (g) qRT-PCR quantification of miR-138-5p in the BALF macrophages in the four abovementioned groups. (h) Macrophages isolated from BALF were subjected to immunoblot analysis with specific antibodies against INOS, Arg-1, EZH2, p-STAT1, PPAR-*γ*, and GAPDH. Expression levels of indicated proteins were quantified by densitometry and normalized with GAPDH. All data are expressed as mean ± SEM. (*N* = 9 − 15/group, ^∗^*P* < 0.05 vs. sham and Sham+circN4bp1-KD group, #*P* < 0.05 vs. CLP+ vector group, determined by one-way ANOVA for multiple group comparisons).

To determine whether circN4bp1 plays a role in the modulation of macrophage actions in inflammation and injury of ARDS, mice were intravenously injected with Si-circN4bp1 lentivirus plasmids to knock down circN4bp1in macrophages (circN4bp1-KD) prior to CLP, followed by measuring the indices of lung injury in the lung 24 h post-CLP. As shown in Figure 7(a), we observed that the circN4bp1-KD-macrophage treated animals displayed a relatively higher long-term survival (65%) compared to the vector group (35%). Furthermore, the expression level of circN4bp1 in the alveolar macrophages was downregulated in the circN4bp1-KD mice comparing with vector-treated CLP mice (Figure 7(b)). The circN4bp1-KD CLP mice also exhibited decreased ALI parameters as evidenced by a reduction of morphological disruption of lung tissue architecture (Figures 7(c) and 7(a)), reduced wet/dry ratio (Figure 7(d)), and BAL protein leakage (Figure 7(e)) in comparison with the vector group.

In order to further evaluate effects and potential molecular mechanism of circN4bp1on macrophage polarization, we next set out to assess the phenotype changes in sepsis-induced ARDS mice of by isolating macrophages from BALF. We noted a remarkably upregulated protein expression of iNOS while downregulated expressions of Arg-1. Furthermore, circN4bp1-KD group mice exhibited significantly lower levels of IL-6 and TNF-*α*, accompanied by higher levels of IL-10 in the macrophages isolated from BALF, relative to sham and vector groups (Figure 7(f)). As we had documented that circN4bp1 promote M1 macrophage polarization through miR-138-5P/EZH2 signaling, we further verify these results in vivo. In consistent with the findings in vitro, we observed a lower expression of miR-138-5p and a higher level of EZH2 in macrophages isolated from the vector ARDS mice in comparison with the sham group; circN4bp1-KD treatment can restore the expression of miR-138-5p but decrease the levels of EZH2 (Figures 7(g) and 7(h)). Besides, we identified that p-STAT1 was significantly inhibited while PPAR-*γ* was activated in the macrophages from BALF of circN4bp1-KD ARDS mice comparing with sham and vector-treated ARDS mice (Figure 7(h)).

### 3.7. Upregulation of circN4bp1 in Macrophages of Sepsis-Induced ARDS Is Partially Attributed to m6A Modification

Recent evidence shows that circRNA is modified by m6A, which affects circRNA levels, and m6A modification could promote macrophage polarization in vitro [18]. To explore the potential mechanism involved in the upregulation of circN4bp1in macrophages of CLP-induced ARDS mice, we first determined the m6A level of circN4bp1 in isolated macrophages by MeRIP-qRT-PCR. As shown in supplementary Figure S4A, the relative m6A level of circN4bp1was remarkably elevated in the macrophages of CLP mice in comparison with sham controls. Next, we examined the relative expression of m6A related genes, including methyltransferase (writer), demethylase (eraser), and reader protein (reader) in macrophages. We found that the mRNA expression levels of METTL3, FTO, and YTHDF2 in CLP mice were obviously elevated compared with control groups, and METTL3 upregulation was the most significant among all these genes (supplementary Figure S4B).

To identify the role of METTL3 in modulating the m6A modification of circN4bp1, qRT-PCR analysis of circN4bp1 levels was conducted in ex vivo LPS-stimulated pulmonary macrophages (MH-S) after METTL3 silencing or treatment with 3-deazaadenosine (DAA), a global methylation inhibitor. Interestingly, the increased circN4bp1was almost reduced to the normal level with knockdown or pharmacological inhibition of METTL3 (supplementary Figure S4C). Moreover, we found two highly conservative m6A sites on circN4bp1 based on the online m6A SRAMP database (http://www.cuilab.cn/sramp) (supplementary Figure S4D). We mutated them and conducted the luciferase reporter assay which showed an augment of the luciferase activity of wild-type vector, but not the mutated vector (supplementary Figure S4E).

## 4. Discussion

In the present study, we showed for the first time that circN4bp1 was overexpressed not only in macrophages of animal models but also in the PBMCs and monocytes from sepsis-induced ARDS patients, and it promoted M1 macrophage activation but inhibited M2 macrophage polarization through the circN4bp1-miR-138-5p-EZH2 axis. Moreover, knockdown of circN4bp1 could alleviate the lung injury and concomitant inflammation in ARDS mice postsepsis by inhibiting M1 macrophage activation. In addition, m6A modifications were critically involved in controlling circN4bp1 expression. Thus, we speculate that circN4bp1 might serve as a potential new therapeutic target for ARDS, adding a new dimension to the functional importance of circRNAs regulation in macrophage polarization in inflammatory state.

Recent findings propose that circRNAs may play a role in regulating the patients' immune system, and misregulation of circRNAs might be an early event in sepsis [19–21]. It is well known that ARDS is one of the major consequences of septic shock [3]. To date, limited clinical research has focused on the potential involvement of circRNAs in the occurrence and development of sepsis induced ARDS. Very recently, a study documented the differential expression of circRNAs in lung tissues of patients with sepsis-induced ARDS and brain dead without ARDS by genome-wide sequencing [22]. However, the clinical evidence concerning the role of circRNAs as diagnostic and prognostic biomarkers in ARDS postsepsis is still lacking. To our knowledge, we are the first to report that the levels of circN4bp1 in both the PBMC and monocytes were significantly increased in sepsis-induced ARDS patients than controls. Besides, a high expression level of circN4bp1 in PBMC and monocytes was noted in ARDS patients who died than those survived, which were strongly associated with high mSOFA scores. Until now, the value of circRNAs in predicting the severity and clinical outcome of ARDS has not been reported. Thus, our study highlights the feasibility of using circN4bp1 as a biomarker for the quick identification of patients who subsequently deteriorate clinically. Furthermore, we identified that silencing of circN4bp1 in macrophages may improve the long-term survival in sepsis-induced ARDS murine animals which are comparable to the clinical observations.

Mounting studies have shown the evidence that circRNAs can function as miRNA sponge via ceRNA crosstalk in various biologic processes, such as cancer, inflammation-related, or autoimmune diseases [5, 6]. We and other studies have previously documented the differential expressed circRNAs in ARDS mice and constructed circRNA-miRNA interaction networks through using bioinformatic tools [11, 23]. However, it is not clear whether those circRNAs can function as ceRNA for the dysregulated miRNAs in ARDS. As the premise of circRNA acing as miRNA sponge is that it is in the cytoplasm, where mature miRNA targets mRNA 3′UTR and inhibits gene expression [6]. Thus, in this study, we used FISH assay to clearly show that circ N4bp1 was a cytoplasmic circRNA. Based on this, our findings further suggested that circN4bp1 might act as a miRNA sponge by interacting with miR-138-5p following miRNA-targeting and cirRIP analyses.

Large number of studies had shown that macrophages are key players for the process of the inflammatory responses in ARDS which tend to shift into the classically activated phenotype (M1) in the acute phase [4, 24, 25]. We further demonstrate that upregulation of circN4bp1 contributes to sepsis-induced ARDS by regulating macrophage polarization and related inflammatory response through the circN4bp1-miR-138-5p ceRNA network. Previous studies had documented that miR-138-5p could inhibit the promoter of the epigenetic regulator EZH2 in cancers, nervous system diseases, and obesity [26–28], and we found that miR-138-5p could interact with the 3′UTR of EZH2 and inhibit its expression, thereby suppressing M1 macrophage differentiation but promoting M2 polarization. These results are in consistent with our previous findings indicating the substantial involvement of EZH2 in macrophage activation in sepsis and ALI [13, 16]. Besides, we observed that circN4bp1 can promote EZH2 expression in macrophage in vitro, while miR-138-5p mimics could partially rescue this effect via circN4bp1/miR-138-5p ceRNA. Moreover, knockdown of circ N4bp1 in macrophages alleviates the inflammation and lung injury in ARDS mice in vivo. We also wondered whether circN4bp1 would affect the signaling pathway involved in macrophage polarization and confirmed that circN4bp1 could regulate macrophage phenotypic shift via STAT1/PPAR*γ* signaling pathway in vivo and in vitro.

Emerging evidence has suggested that m6A modification is a gene regulatory mechanism involved in the modulation of RNA stability, localization, splicing, and translation [29]. However, the function and mechanism of m6A modification in ARDS have not yet been reported. Interestingly, we observed the m6A level of circN4bp1 was unregulated in macrophages from the ARDS mice model. It is well known that m6A modification is a dynamic and reversible process, including m6A writers (METTL3 and METTL14), erasers (FTO and ALKBH5), and readers (YTHDC1/2, YTHDF1/2/3 and IGF2BP1/2/3) [29, 30]. We further found that METTL3, FTO, and YTHDF2 were highly expressed in macrophages; among them, the expression of METTL3 showed the most significant upregulation. Notably, knockdown or pharmacological inhibition of METTL3 could effectively block the increased circN4bp1 caused by LPS stimulation in pulmonary macrophages, suggesting that m6A modification is critical for the regulation of expression of circN4bp1 in macrophages. Future investigations are required to delineate the mechanisms by which m6A modifications regulates circRNAs in ARDS and test the possibilities of regulating m6A methylation of circRNAs to treat ARDS.

Collectively, this investigation suggests a potential mechanism by which circN4bp1 targets macrophage polarization and function in sepsis-induced ARDS by sponging miR-138-5p, thereby promotion of the expression of EZH2. In addition, the upregulation of circN4bp1might be modified by m6A methylation. These results suggest that circN4bp1 may be a biomarker and potential therapeutic target for preventing and treating of ARDS induced by sepsis.

## Data Availability

All data generated or analyzed during this study are included in this published article [and its supplementary information files].

## Abbreviations

ALI: Acute lung injury
ARDS: Acute respiratory distress syndrome
CLP: Cecal ligation and puncture
circRIP: circRNA immunoprecipitation
ICU: Intensive care unit
FISH: Fluorescence in situ hybridization
BMDMs: Marrow-derived macrophages
mSOFA: Modified Sequential Organ Failure Assessment
m6A: N6-Methyladenosine
ncRNAs: Noncoding RNAs
PBMC: Peripheral blood mononuclear cells
qRT-PCR: Quantitative real-time PCR
siRNAs: Small interfering RNAs.

## Supplementary Materials

Supplementary Materials Table S1: clinical characteristics of the sepsis-induced ARDS patients and healthy control. Table S2: details of primers used for RT-PCR. Table S3: details of primary antibodies used for immunoblotting analysis. Figure S1: MH-S was transfected with Si-circN4bp1 (circN4bp1-KD), circN4bp1 lentivirus plasmids (circN4bp1-OE), or scrambled control and then exposed to either LPS (50 ng/ml) or IL-4 (10 ng/ml) for an additional 24 h. The expressions of iNOS, Arg-1, p-STAT1, and PPAR-*γ* were quantified by western blot and IL-6, and TNF-*α* and IL-10 were measured by ELISA. Figure S2: RAW264.7 and MH-S were transfected with miR-138-5p mimic or inhibitor and then exposed to either LPS (50 ng/ml) or IL-4 (10 ng/ml) for an additional 24 h. The levels of IL-6, TNF-*α*, and IL-10 were quantified by ELISA. Figure S3: MH-S cells was transfected with miR-138-5p mimic with/without circN4bp1 lentivirus plasmids (circN4bp1-OE) or scrambled control and then exposed to either LPS (50 ng/ml) or IL-4 (10 ng/ml) for an additional 24 h. The levels of IL-6, TNF-*α*, and IL-10 were quantified by ELISA. The expressions of iNOS and Arg-1 were quantified by western blot.
