# Supplementary material for: CircN4bp1 Facilitates Sepsis-Induced Acute Respiratory Distress Syndrome through Mediating Macrophage Polarization via the miR-138-5p/EZH2 Axis
Source: Mediators Inflamm. 2021 Dec 30;2021:7858746. doi: 10.1155/2021/7858746 (PMC8739551; doi:10.1155/2021/7858746)
Supplement: Supplementary Materials — Table S1: clinical characteristics of the sepsis-induced ARDS patients and healthy control. Table S2: details of primers used for RT-PCR. Table S3: details of primary antibodies used for immunoblotting analysis. Figure S1: MH-S was transfected with Si-circN4bp1 (circN4bp1-KD), circN4bp1 lentivirus plasmids (circN4bp1-OE), or scrambled control and then exposed to either LPS (50 ng/ml) or IL-4 (10 ng/ml) for an additional 24 h. The expressions of iNOS, Arg-1, p-STAT1, and PPAR-γ were quantified by western blot and IL-6, and TNF-α and IL-10 were measured by ELISA. Figure S2: RAW264.7 and MH-S were transfected with miR-138-5p mimic or inhibitor and then exposed to either LPS (50 ng/ml) or IL-4 (10 ng/ml) for an additional 24 h. The levels of IL-6, TNF-α, and IL-10 were quantified by ELISA. Figure S3: MH-S cells was transfected with miR-138-5p mimic with/without circN4bp1 lentivirus plasmids (circN4bp1-OE) or scrambled control and then exposed to either LPS (50 ng/ml) or IL-4 (10 ng/ml) for an additional 24 h. The levels of IL-6, TNF-α, and IL-10 were quantified by ELISA. The expressions of iNOS and Arg-1 were quantified by western blot. [file 7858746.f1.zip › Supplementary material -Figure S2 (1).docx]

**Figure S2**


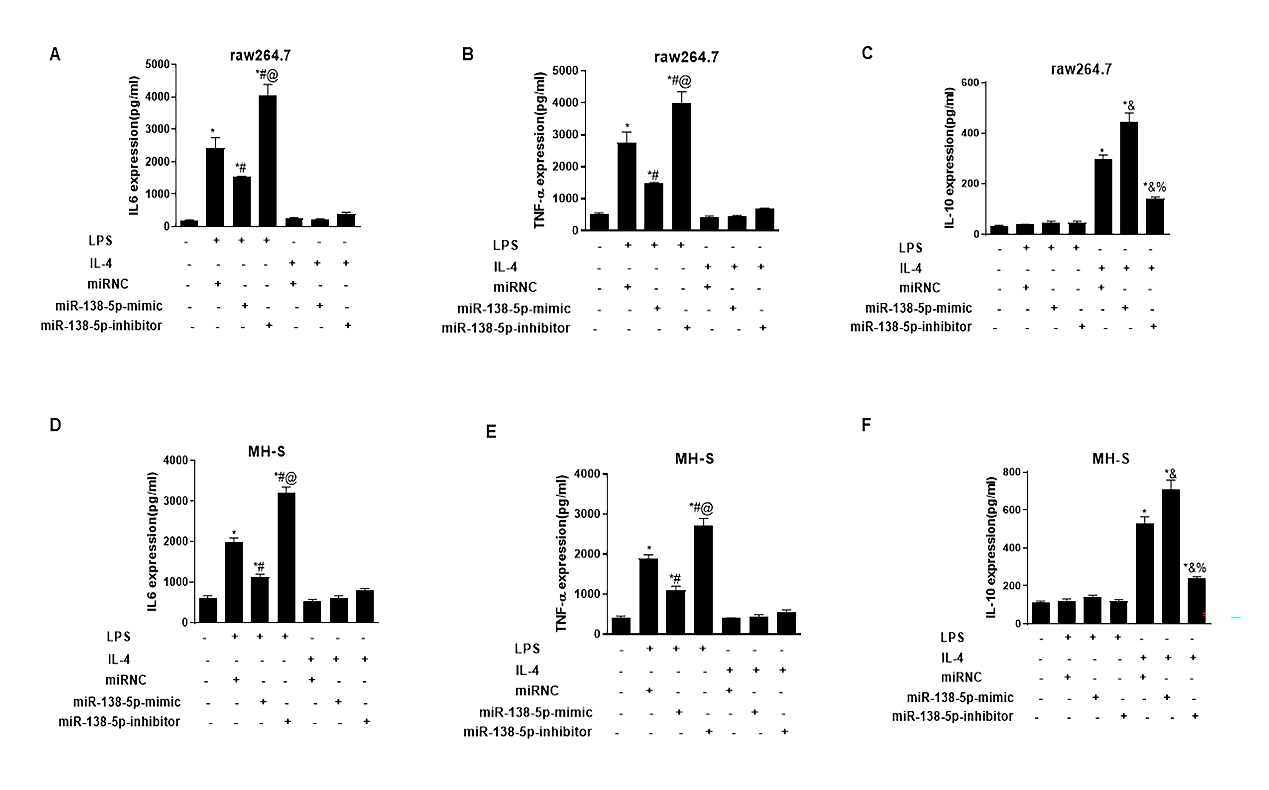


RAW264.7 and MH-S were transfected with miR-138-5p mimic or inhibitor and then exposed to either LPS (50 ng/ml) or IL-4 (10ng/ml) for an additional 24 h. The levels of IL-6(A, D), TNF-α (B, E) were measured by ELISA in the supernatants of LPS stimulated raw264.7 or MH-S cells and IL-10 (C, F) levels were qualified by ELISA in IL-4 stimulated raw264.7 cells or MH-S cells. All data are expressed as mean ± SEM. (* *p*﹤0.05 vs. NC group, *^#^p*﹤0.05 vs. LPS stimulated group, ^@^ *p*﹤0.05 vs. miR-138-5p mimic +LPS group, & *p*﹤0.05vs. IL-4 stimulated group and % *p*﹤0.05 vs. miR-138-5p mimic +IL-4 group determined by one-way ANOVA for multiple group comparisons).
