# Supplementary material for: CircN4bp1 Facilitates Sepsis-Induced Acute Respiratory Distress Syndrome through Mediating Macrophage Polarization via the miR-138-5p/EZH2 Axis
Source: Mediators Inflamm. 2021 Dec 30;2021:7858746. doi: 10.1155/2021/7858746 (PMC8739551; doi:10.1155/2021/7858746)
Supplement: Supplementary Materials — Table S1: clinical characteristics of the sepsis-induced ARDS patients and healthy control. Table S2: details of primers used for RT-PCR. Table S3: details of primary antibodies used for immunoblotting analysis. Figure S1: MH-S was transfected with Si-circN4bp1 (circN4bp1-KD), circN4bp1 lentivirus plasmids (circN4bp1-OE), or scrambled control and then exposed to either LPS (50 ng/ml) or IL-4 (10 ng/ml) for an additional 24 h. The expressions of iNOS, Arg-1, p-STAT1, and PPAR-γ were quantified by western blot and IL-6, and TNF-α and IL-10 were measured by ELISA. Figure S2: RAW264.7 and MH-S were transfected with miR-138-5p mimic or inhibitor and then exposed to either LPS (50 ng/ml) or IL-4 (10 ng/ml) for an additional 24 h. The levels of IL-6, TNF-α, and IL-10 were quantified by ELISA. Figure S3: MH-S cells was transfected with miR-138-5p mimic with/without circN4bp1 lentivirus plasmids (circN4bp1-OE) or scrambled control and then exposed to either LPS (50 ng/ml) or IL-4 (10 ng/ml) for an additional 24 h. The levels of IL-6, TNF-α, and IL-10 were quantified by ELISA. The expressions of iNOS and Arg-1 were quantified by western blot. [file 7858746.f1.zip › Supplementary material -Figure S4 (1).docx]

**Figure S4**


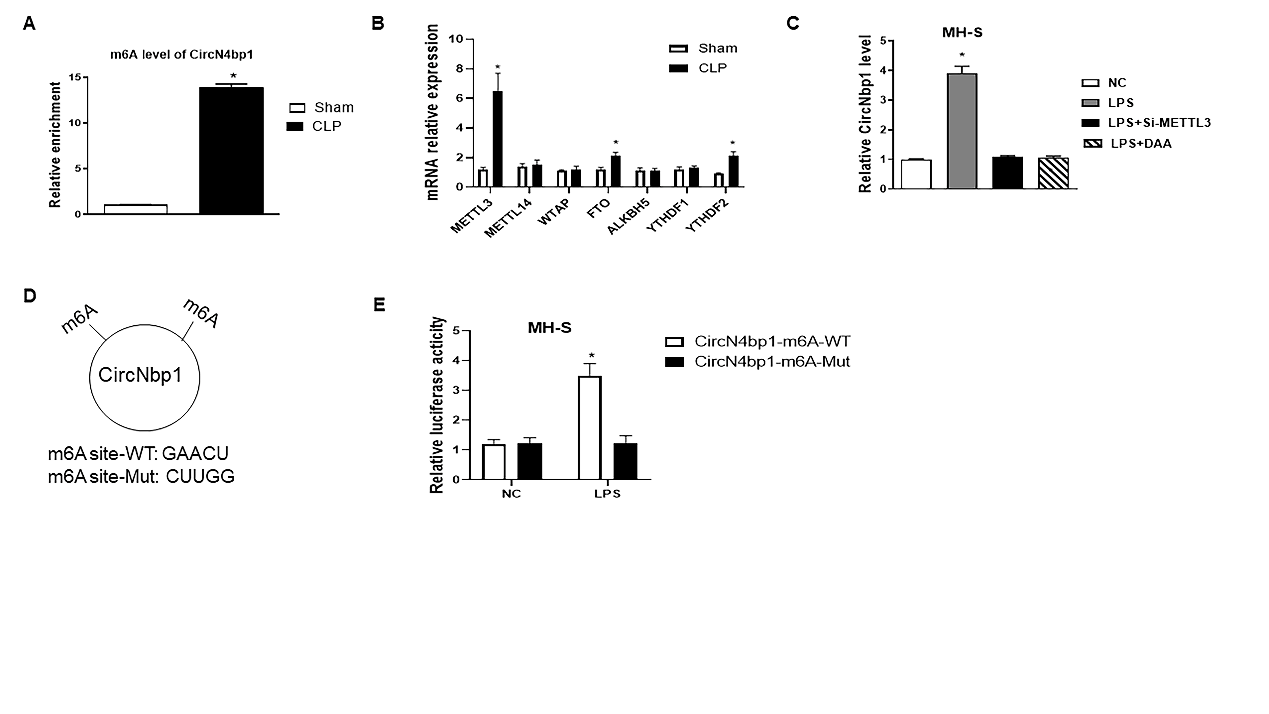


(A) MeRIP-PCR analysis of m6A enrichment of circN4bp1 in the pulmonary macrophages of ARDS mice and control mice. (B) qRT-PCR analysis of m6A related genes in pulmonary macrophages of ARDS mice and control mice. All data are expressed as mean ± SEM. * p <0.05 VS. Sham group. (C) qRT-PCR analysis of circN4bp1 level in LPS-stimulated pulmonary macrophages cells (MH-S) after METTL3 silencing or DAA treatment. * p <0.05 VS. LPS group. (D) The cartoon showing two m6A sites on circN4bp1. (E) Luciferase reporter assay in MH-S cells co-transfected with wild-type or mutant circN4bp1 reporter and exposed to either LPS (50 ng/ml) or not. * p <0.05 VS. circN4bp1-m6A-WT group.
