# Supplementary material for: CircN4bp1 Facilitates Sepsis-Induced Acute Respiratory Distress Syndrome through Mediating Macrophage Polarization via the miR-138-5p/EZH2 Axis
Source: Mediators Inflamm. 2021 Dec 30;2021:7858746. doi: 10.1155/2021/7858746 (PMC8739551; doi:10.1155/2021/7858746)
Supplement: Supplementary Materials — Table S1: clinical characteristics of the sepsis-induced ARDS patients and healthy control. Table S2: details of primers used for RT-PCR. Table S3: details of primary antibodies used for immunoblotting analysis. Figure S1: MH-S was transfected with Si-circN4bp1 (circN4bp1-KD), circN4bp1 lentivirus plasmids (circN4bp1-OE), or scrambled control and then exposed to either LPS (50 ng/ml) or IL-4 (10 ng/ml) for an additional 24 h. The expressions of iNOS, Arg-1, p-STAT1, and PPAR-γ were quantified by western blot and IL-6, and TNF-α and IL-10 were measured by ELISA. Figure S2: RAW264.7 and MH-S were transfected with miR-138-5p mimic or inhibitor and then exposed to either LPS (50 ng/ml) or IL-4 (10 ng/ml) for an additional 24 h. The levels of IL-6, TNF-α, and IL-10 were quantified by ELISA. Figure S3: MH-S cells was transfected with miR-138-5p mimic with/without circN4bp1 lentivirus plasmids (circN4bp1-OE) or scrambled control and then exposed to either LPS (50 ng/ml) or IL-4 (10 ng/ml) for an additional 24 h. The levels of IL-6, TNF-α, and IL-10 were quantified by ELISA. The expressions of iNOS and Arg-1 were quantified by western blot. [file 7858746.f1.zip › Supplementary material -Table S1 (1).docx]

**Table S1**

**Clinical characteristics of the sepsis-induced ARDS patients and healthy control**

|  | ARDS patients(n=40) | Healthy control(n=40) | P value |
| --- | --- | --- | --- |
| **Baseline** |  |  |  |
| Age, median (IQR), y | 56 (35-67) | 58 (30-65) | 0.067 |
| Male, n (%) | 28（70%） | 26（65%） | 0.211 |
| Sepsis etiology, No. (%) |  |  |  |
| Thorax | 26 (65) |  |  |
| Abdomen | 9 (22.5) |  |  |
| Urinary tract | 3 (7.5) |  |  |
| Central nervous system | 1 (2.5) |  |  |
| Central venous catheter | 1 (2.5) |  |  |
| Admission source, No. (%) |  |  |  |
| Emergency department | 28 (70) |  |  |
| Outside hospital transfer | 6 (15) |  |  |
| Inpatient ward transfer | 4 (10) |  |  |
| Operating room | 2 (5) |  |  |
| Respiratory, mean (SD) |  |  |  |
| Tidal volume, ml | 412.7 (86.4) |  |  |
| PaO2/FiO2 ratio at baseline | 178.5 (92.1) |  |  |
| PEEP, cmH2O | 8.5 (4.2) |  |  |
| Oxygenation index, mean (SD) | 10.8 (9.2) |  |  |
| Incidence of shock, No (%) |  |  |  |
| At baseline, vasopressor in use | 26 |  |  |
| mSOFA scores, mean (SD) | 8.7 (3.0) |  |  |
| **Outcome measures** |  |  |  |
| Total hospital time, median (IQR) | 14（8.25-21） |  |  |
| ICU time, median (IQR) | 10（5.75-14.25） |  |  |
| 28-Day mortality, No. (%) | 17 (42.5) |  |  |

Data are presented as frequencies and percentages (%) for categorical data and medians and interquartile ranges [IQR] for continuous variables. P-values were calculated using Chi-Square tests for categorical data and one-way ANOVA on log-transformed continuous data. PEEP, positive end-expiratory pressure; mSOFA, modified Sequential Organ Failure Assessment; Oxygenation index = mean airway pressure × Fio_2_/Pao_2_; mSOFA score range, 0 to 20 (values >8 indicate severe illness); ICU=intensive care unit.
