# Supplementary material for: CircN4bp1 Facilitates Sepsis-Induced Acute Respiratory Distress Syndrome through Mediating Macrophage Polarization via the miR-138-5p/EZH2 Axis
Source: Mediators Inflamm. 2021 Dec 30;2021:7858746. doi: 10.1155/2021/7858746 (PMC8739551; doi:10.1155/2021/7858746)
Supplement: Supplementary Materials — Table S1: clinical characteristics of the sepsis-induced ARDS patients and healthy control. Table S2: details of primers used for RT-PCR. Table S3: details of primary antibodies used for immunoblotting analysis. Figure S1: MH-S was transfected with Si-circN4bp1 (circN4bp1-KD), circN4bp1 lentivirus plasmids (circN4bp1-OE), or scrambled control and then exposed to either LPS (50 ng/ml) or IL-4 (10 ng/ml) for an additional 24 h. The expressions of iNOS, Arg-1, p-STAT1, and PPAR-γ were quantified by western blot and IL-6, and TNF-α and IL-10 were measured by ELISA. Figure S2: RAW264.7 and MH-S were transfected with miR-138-5p mimic or inhibitor and then exposed to either LPS (50 ng/ml) or IL-4 (10 ng/ml) for an additional 24 h. The levels of IL-6, TNF-α, and IL-10 were quantified by ELISA. Figure S3: MH-S cells was transfected with miR-138-5p mimic with/without circN4bp1 lentivirus plasmids (circN4bp1-OE) or scrambled control and then exposed to either LPS (50 ng/ml) or IL-4 (10 ng/ml) for an additional 24 h. The levels of IL-6, TNF-α, and IL-10 were quantified by ELISA. The expressions of iNOS and Arg-1 were quantified by western blot. [file 7858746.f1.zip › Supplementary material -Table S2 (1).docx]

**Table S2. Details of primers used for RT-PCR**

| **Gene** | **forward** | **reverse** |
| --- | --- | --- |
| INOS | 5’-GGAGCCAGCTCTGCATTATC-3’ | 5’-TTTTTGTCTCCAAGGGACCAG-3’ |
| Arg-1 | 5’-CAGAAGAATGGAAGAGTCAG-3’ | 5’-CAGATATGCAGGGAGTCAC-3’ |
| FIZZ-1 | 5’-TCCCAGTGAATACTGATGAGA-3’ | 5’-CCACTCTGGATCTCCCAAGA-3’ |
| circN4bp1 | 5’-TGCGGAAATTAGGGTCGGAA-3’ | 5’-CCGACCGGAACTTGAGTCTT-3’ |
| miR-138-5p | 5’-GCTTAAGGCACGCGG-3’ | 5’-GTGCAGGGTCCGAGG-3’ |
| METTL3 | 5’-CTATCTCCTGGCACTCGCAAGA-3’ | 5’-GCTTGAACCGTGCAACCACATC-3’ |
| FTO | 5’-CCAGAACCTGAGGAGAGAATGG-3’ | 5’-CGATGTCTGTGAGGTCAAACGG-3’ |
| YTHDF2 | 5’-TAGCCAGCTACAAGCACACCAC-3’ | 5’-CAACCGTTGCTGCAGTCTGTGT-3’ |
| METTL14 | 5’-CTGAAAGTGCCGACAGCATTGG-3' | 5' -CTCTCCTTCATCCAGATACTTACG-3' |
| WTAP | 5’-GCCAACTGCTGGCGTGTCT-3' | 5’-ATGGCGAAGTGTCGAATGCT-3' |
| ALKBH5 | 5’-CCAGCTATGCTTCAGATCGCCT-3' | 5’-GGTTCTCTTCCTTGTCCATCTCC-3' |
| YTHDF1 | 5’-CAAGCACACAACCTCCATCTTCG-3' | 5’-GTAAGAAACTGGTTCGCCCTCAT-3' |
| GAPDH | 5’-GGATTGGTCGTATTGGG-3’ | 5’-GGAAGATGGTGATGGGATT-3’ |
