# Supplementary material for: CircN4bp1 Facilitates Sepsis-Induced Acute Respiratory Distress Syndrome through Mediating Macrophage Polarization via the miR-138-5p/EZH2 Axis
Source: Mediators Inflamm. 2021 Dec 30;2021:7858746. doi: 10.1155/2021/7858746 (PMC8739551; doi:10.1155/2021/7858746)
Supplement: Supplementary Materials — Table S1: clinical characteristics of the sepsis-induced ARDS patients and healthy control. Table S2: details of primers used for RT-PCR. Table S3: details of primary antibodies used for immunoblotting analysis. Figure S1: MH-S was transfected with Si-circN4bp1 (circN4bp1-KD), circN4bp1 lentivirus plasmids (circN4bp1-OE), or scrambled control and then exposed to either LPS (50 ng/ml) or IL-4 (10 ng/ml) for an additional 24 h. The expressions of iNOS, Arg-1, p-STAT1, and PPAR-γ were quantified by western blot and IL-6, and TNF-α and IL-10 were measured by ELISA. Figure S2: RAW264.7 and MH-S were transfected with miR-138-5p mimic or inhibitor and then exposed to either LPS (50 ng/ml) or IL-4 (10 ng/ml) for an additional 24 h. The levels of IL-6, TNF-α, and IL-10 were quantified by ELISA. Figure S3: MH-S cells was transfected with miR-138-5p mimic with/without circN4bp1 lentivirus plasmids (circN4bp1-OE) or scrambled control and then exposed to either LPS (50 ng/ml) or IL-4 (10 ng/ml) for an additional 24 h. The levels of IL-6, TNF-α, and IL-10 were quantified by ELISA. The expressions of iNOS and Arg-1 were quantified by western blot. [file 7858746.f1.zip › Supplementary material -Table S3 (1).docx]

**Table S3. Details of primary antibodies used for immunoblotting analysis**

| **Antibody** | **Catalogue number** | **Supplier** | **Dilution** |
| --- | --- | --- | --- |
| EZH2 | #5246 | Cell Signaling Technology (Danvers, MA, USA) | 1:1000 |
| iNOS | #:2982 | Cell SignalingTechnology | 1:1000 |
| Arginase-1 | #79404 | Cell Signaling Technology | 1:1000 |
| p-STAT1 | #8217 | Cell SignalingTechnology | 1:1000 |
| PPAR-γ | #2435 | Cell SignalingTechnology | 1:1000 |
| GAPDH | sc-32233 | Santa Cruz Biotechnology (Santa Cruz, CA, USA) | 1:1000 |
